# Mechanisms Underlying Neurodegenerative Disorders and Potential Neuroprotective Activity of Agrifood By-Products

**DOI:** 10.3390/antiox12010094

**Published:** 2022-12-30

**Authors:** Cristina Angeloni, Marco Malaguti, Cecilia Prata, Michela Freschi, Maria Cristina Barbalace, Silvana Hrelia

**Affiliations:** 1 Department for Life Quality Studies, Alma Mater Studiorum–University of Bologna, Corso D’Augusto 237, 47921 Rimini, Italy; 2 Department of Pharmacy and Biotechnology, Alma Mater Studiorum–University of Bologna, Via Irnerio 48, 40126 Bologna, Italy

**Keywords:** agrifood by-products, grape, coffee, tomato, olive, chestnut, onion, apple, pomegranate, neurodegeneration

## Abstract

Neurodegenerative diseases, characterized by progressive loss in selected areas of the nervous system, are becoming increasingly prevalent worldwide due to an aging population. Despite their diverse clinical manifestations, neurodegenerative diseases are multifactorial disorders with standard features and mechanisms such as abnormal protein aggregation, mitochondrial dysfunction, oxidative stress and inflammation. As there are no effective treatments to counteract neurodegenerative diseases, increasing interest has been directed to the potential neuroprotective activities of plant-derived compounds found abundantly in food and in agrifood by-products. Food waste has an extremely negative impact on the environment, and recycling is needed to promote their disposal and overcome this problem. Many studies have been carried out to develop green and effective strategies to extract bioactive compounds from food by-products, such as peel, leaves, seeds, bran, kernel, pomace, and oil cake, and to investigate their biological activity. In this review, we focused on the potential neuroprotective activity of agrifood wastes obtained by common products widely produced and consumed in Italy, such as grapes, coffee, tomatoes, olives, chestnuts, onions, apples, and pomegranates.

## 1. Introduction

Advances in medical science have resulted in a considerable increase in human life expectancy. Alongside this extremely positive result, there is a consequent dizzying increase in age-related non-communicable diseases such as cognitive decline and neurodegenerative disorders [1]. These disorders, including Parkinson’s disease (PD), Huntington’s disease (HD), amyotrophic lateral sclerosis (ALS), and Alzheimer’s disease (AD), are characterized by the progressive loss in selected areas of the nervous system, which determine clinical outcomes, such as motor impairment and/or cognitive decline. These diseases have a significant impact not only on patients’ quality of life but also on the public health cost and on the social burden. In spite of their diverse clinical manifestations, neurodegenerative diseases share common features and mechanisms, such as abnormal protein misfolding and aggregation, oxidative stress, inflammation, mitochondrial dysfunctions, impaired Ca^2+^ homeostasis, excitotoxicity, and apoptosis [2,3,4,5]. These mechanisms are interconnected in a complex network that ultimately leads to cellular dysfunction and death.

Despite a great number of ongoing investigations, pharmacological options to treat neurodegenerative conditions are limited, so there is an urgent need to identify alternative strategies to prevent, slow and even stop neurodegeneration. Perhaps, the multifactorial nature of neurodegeneration led to the failure of the different drug therapies that in the last years have been developed following the paradigm of “one drug, one activity, one disease”. However, this idea changed through the discovery of multi-target bioactive compounds, in which the same molecule can exert its effects by targeting different molecular pathways, opening a new avenue for research. From this point of view, a wide variety of molecules derived from natural products have been evaluated for their multiple biological effects, such as the compounds found in the Mediterranean diet rich in cereals, extra virgin olive oil, legumes, fruit, and vegetables [6,7].

The agrifood industries are among the largest producers of waste in the world [8]. Estimates indicate that every year around 90 million tons of food by-products are generated in the European Union, with an extremely negative impact on the environment [9]. An amount of 8–10% of greenhouse gas emissions, 23% of fertilizer consumption, and 25% of the freshwater used in agriculture is attributable to this waste [10].

However, it is now widely known that agrifood by-products are still rich in valuable bioactive compounds [11,12,13]. Consequently, in recent years, researchers have been dedicated to the development of approaches to recycling several agrifood wastes such as peel, leaves, seeds, bran, kernel, pomace, and oil cake [14], which are a potential source of antioxidant compounds, vitamins, minerals, along with dietary fiber, essential fatty acids, oligosaccharides, and oligopeptides [15,16]. In recent years, the health-promoting properties of different agrifood wastes have been investigated, highlighting different biological activities such as antioxidant, anti-inflammatory, and chemopreventive [17,18,19,20].

In this review, we focused on the potential neuroprotective activity of agrifood wastes, all of plant origin, obtained by common products widely produced and consumed in Italy, such as grapes, wine, coffee, tomatoes, olive oil, chestnuts, onions, apples, and pomegranates.

## 2. Literature Search Strategy

The literature search was conducted on the scientific literature database PubMed. The search strategy combined the descriptors using the Booleans operators (AND/OR) in the following way: (coffee OR chestnut OR wine OR grape OR “olive oil” OR olive OR tomato OR onion OR pomegranate OR apple) AND (waste OR “by-products”) AND (neuro* OR inflammation OR “oxidative stress” OR “mitochondrial dysfunction”). Two hundred seventy-five papers were retrieved. Only peer-reviewed papers written in English and available in the full text were selected. Since not all the papers precisely addressed the topics covered by the review, a selection was independently carried out by the six authors, and potential conflicts were resolved through confrontation and discussion.

## 3. Molecular Mechanisms Implicated in Neurodegeneration

Neurodegeneration is a complex process with multifactorial etiology, primarily affecting neurons of the human brain associated with the progressive loss of neural tissues, including neuron death [21]. It is a condition closely related to aging, and one of its main hallmarks is an impairment of protein homeostasis that leads to the accumulation of insoluble deposits and inclusion bodies in different areas of the brain [22,23]. Many other pathological mechanisms have been linked to neurodegeneration, including oxidative stress, neuroinflammation, mitochondrial dysfunction, calcium deregulation, axonal transport deficits, and DNA damage [24,25,26]. The persistence of these conditions gradually overwhelms self-defense mechanisms inducing life-death imbalances and culminating in programmed cell death through several death pathways, including apoptosis, necrosis, autophagy, and parthanatos [27].

**Figure 1:**
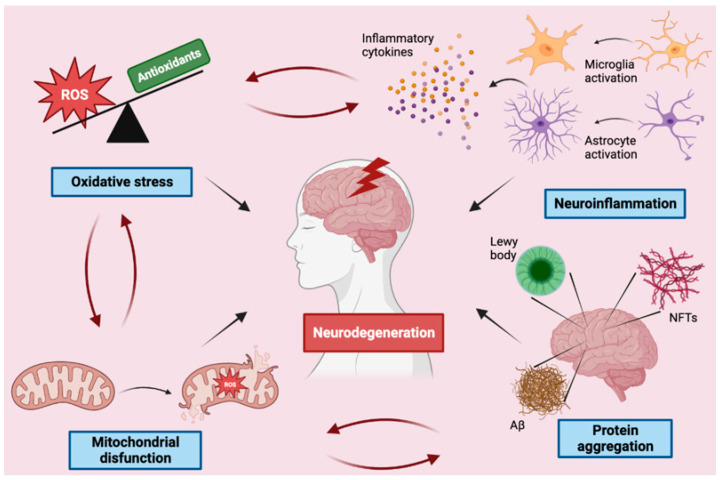
Schematic representation of the main mechanisms involved in neurodegeneration. ROS—reactive oxygen species, NFTs—neurofibrillary tangles.

In the following paragraphs, the main molecular mechanisms of neurodegeneration will be addressed (Figure 1).

### 3.1. Protein Aggregation

Misfolded proteins and their gradual accumulation and aggregation are common hallmarks of different neurodegenerative conditions and are believed to be the main cause of these disorders [28,29,30]. Aggregation is usually triggered by a seed or/and an oligomer, in which a non-native conformation is adopted because of the establishment of interactions among specific elements of the misfolded protein. This process can then spread to other proteins that are converted into toxic forms. In general, misfolded proteins produce intermolecular structures rich in β sheets that are generated when soluble oligomers begin to assemble into small protofibrils [31,32]. Extracellular or intracellular inclusions are formed as a consequence of protein aggregation.

The various neurodegenerative diseases are characterized by the presence of different types of protein aggregates. For example, β-amyloid plaques are a hallmark of Alzheimer’s disease; Tau, a microtubule-associated protein, represents another misfolded protein commonly found in diseases such as Alzheimer’s, Parkinson’s, and Huntington’s diseases; the presence of α-synuclein pathological inclusions is typical of Parkinson’s disease and other disorders, including Lewy body dementia, Lewy body variant of Alzheimer’s disease, and multisystem atrophy; TAR DNA-binding protein 43 (TDP-43) is observed in ALS and in frontotemporal dementia [33,34,35,36,37].

In particular, Aβ is synthesized from the transmembrane neuronal amyloid precursor protein (APP) by the sequential action of two enzymes: β- and γ-secretase [38]. There are two main variants of Aβ: Aβ42 consisting of 42 residues, and Aβ40 consisting of 40 residues. The only difference between Aβ42 and Aβ40 is that Aβ42 has two other residues at the C-terminus. Aβ monomers interact, forming different types of aggregates, including oligomers, protofibrils, and amyloid fibrils. Amyloid fibrils are larger and insoluble and can further assemble into amyloid plaques, while amyloid oligomers are soluble and can spread throughout the brain [39]. In the Alzheimer’s brain, these aggregates consist mainly of Aβ42 [40,41]. More and more studies suggest that oligomers, rather than fibers, are the main species that cause toxicity and are involved in the seeding and spreading of the disease [42,43]. Aβ accumulation has important consequences, such as the generation of ROS and the activation of the inflammatory pathway mediated by Nf-kB [29,44,45], establishing a dangerous vicious circle that leads to mitochondrial dysfunction, cognitive deterioration, and culminates in neurodegeneration [41].

Tau protein, a microtubule-associated protein, is another misfolded protein commonly found in neurodegenerative diseases. In humans, there are six different isoforms of Tau ranging from 50 to 70 kDa; in the adult brain, all six isoforms are present, while the fetal brain contains only the shortest one [46]. Tau contains many phosphorylation sites that can be phosphorylated by approximately 20 different kinases [41]. Under physiological conditions, Tau is an axonal protein whose main role is to promote microtubule stability [47]. This function is also known as microtubule-associated protein (MAP). However, the abnormal hyperphosphorylation of Tau reduces its affinity for the microtubules and makes the Tau protein susceptible to aggregation into the so-called neurofibrillary tangles (NFTs) [48]. NFTs destabilize microtubules damaging neurons’ cytoskeleton and transport system [49]. Accumulation of Tau has been observed to increase with age and is related to cognitive decline, forebrain atrophy, and neuronal loss in the hippocampus and neocortex regions of the brain [50]. The amount of Tau in the brain is thought to increase about 10–15 years before symptoms appear [51].

α-synuclein is considered the main pathological protein in different neurodegenerative disorders. It is a small protein of 140 amino acids with an intrinsically disordered structure, coded by the SNCA (synuclein) gene, and mainly expressed in CNS [52]. It is called “synuclein” because it is expressed in the nuclear envelope and was first identified in synapses [53]. Although its abundance in the brain, to date, the α-synuclein-specific function has not been fully elucidated [13,54]. α-synuclein contains three main regions: the N-terminal domain, formed by the first 60 residues, which has the α-helix propensity and an amphipathic membrane binding capacity [38]. The carboxyl-terminal (C-terminal) domain, composed of residues 96–140, is the segment where major phosphorylation and truncation occur. The central domain, encompassing residues 61–95, is known as the non-amyloid component (NAC) and contains a highly hydrophobic motif. This region is highly amyloidogenic and responsible for the formation and aggregation of insoluble protofibrils and fibrils, which, in association with other molecules such as ubiquitin, neurofilament protein, alpha B crystallin, organelles, and lipid membranes, forms Lewy bodies [55,56]. Increasing scientific evidence suggests that abnormal α-synuclein aggregation in intracellular insoluble protein inclusions in the brain is involved in the pathophysiology of several neurodegenerative diseases, including Parkinson’s disease, atrophy multisystem disease and dementia with Lewy bodies [57].

The TDP-43 protein is a peptide formed by about 400 amino acids, which are mainly found in the nucleus, but in cases of neurodegenerative TDP-43 proteinopathies, it is present as cytoplasmic aggregates, presumably containing post-translational modifications [58]. TDP-43 is a key determinant in RNA regulations, including RNA splicing and mRNA stability involved in neural development [59]. In 2006, for the first time, two important studies identified hyperphosphorylated and ubiquitinated cytoplasmic TDP-43 in anterior horn motor neurons of ALS patients [60,61]. The presence of these insoluble hyperphosphorylated TDP-43 cytoplasmic inclusions is now considered a key pathological hallmark of ALS and frontotemporal dementia [62,63]. Cytoplasmic TDP-43 aggregation plausible appears via a gradual process involving the development of sequential disordered intermediate species, including misfolded and oligomeric TDP-43, a process which likely leads to the subsequent deposition of large, insoluble TDP-43 inclusions [64]. It has been suggested that different pathological mechanisms contribute to their generation, exert varying biochemical properties, and have independent effects on neuronal function and viability [64,65,66].

To counteract the formation of protein aggregates, human cells have developed a network of complex mechanisms aimed at maintaining protein homeostasis or proteostasis. Among these, molecular chaperones contribute to proteostasis by facilitating the folding and disaggregation of proteins [67] and the proteasome and autophagic systems that eliminate toxic misfolded proteins [68]. Given the importance of these cell systems in maintaining cell integrity, understanding how these components are regulated in neurodegeneration could open a new avenue of intervention strategies for the treatment of neurodegenerative diseases.

### 3.2. Neuroinflammation

Inflammation is an immune defense mechanism of the host against infections, trauma, accumulation of toxins and other pathological lesions [69,70]. Neuroinflammation, in particular, is the inflammatory response that develops in the central nervous system (CNS) and is a very complex process that involves different types of cells, including microglia, astrocytes, oligodendrocytes that act in a concerted and synergistic way. These cells interact in a coordinated manner thanks to neurotransmitters, ions, neurotrophic factors, and cytokines. Among these cells, microglia and astrocytes are the main mediators of neuroinflammation. Under normal conditions, microglia cells, similarly to macrophages, contribute to the maintenance of brain homeostasis and perform various neuronal reparative functions such as the elimination of dendritic debris, synaptic organization, the biological response to biotoxins, and the phagocytosis of abnormal proteins [71]. For example, in the first stage of AD, microglia can protect the brain from neurodegeneration through phagocytic clearance of β-amyloid (Aβ) [72]. Activated microglia promote the proliferation of astrocytes inducing a neuroprotective effect and the repair of damaged neuronal tissue [73,74]. Activated astrocytes present morphological and biochemical characteristics: cell hypertrophy, positive regulation of intermediate filaments, and increased cell proliferation and motility in response to brain injury [73]. Despite the fact that neuroinflammation is a fundamental protective mechanism, excessive or prolonged neuroinflammation can negatively induce deleterious damage to the brain [75,76]. Aging, metabolic diseases, and viral infections are among the risk factors that can induce chronic neuroinflammation leading to neurodegeneration [77]. Neuroinflammation is characterized by the activation of Nuclear Factor Kappa B (NF-κB), a transcription factor that modulates the expression of different genes involved in inflammation, apoptotic cell death, cell survival, and neuronal differentiation.

Activated microglia can be classified into two opposite phenotypes: classic M1, proinflammatory, or alternative M2, anti-inflammatory, following the paradigm used for macrophages [78,79,80]. The simultaneous activation of signaling pathways mediated by TLR and IFN-γ activates the classic M1 proinflammatory phenotype [81,82] characterized by the production of proinflammatory cytokines and chemokines, responsible for tissue damage [81,83]. It is through the activation of Nf- κB that the microglia induces the upregulation of inflammatory genes such as iNOS and COX-2, and proinflammatory mediators such as TNF-α, interleukin-6 (IL-6), interleukin-1 (IL-1), reactive oxygen species (ROS), and BACE1 [79,84,85]. M1 microglia also over-express several enzymes, such as NADPH oxidase, responsible for the production of superoxide anion, and inducible nitric oxide synthase. The M1 phenotype is characterized by an inhibition of phagocytic activity [82,86] and has a negative effect on the differentiation and maturation of neural progenitor cells on the formation of synapses and plasticity.

In contrast, the M2 neuroprotective microglia can be activated by IL-4, IL-10, IL-13, and transforming growth factor-β (TGF-β), which trigger the production of diverse factors, including FIZZ1, Chitinase-3-Like-3 (Chi3l3), Arginase 1, Ym1, CD206, insulin-like growth factor 1 (IGF-1), and Frizzled class receptor 1 (Fzd1) [83,87,88]. These factors from microglia may be associated with increased phagocytic capacity, synaptic communication, plasticity and neuronal repair [78,80]. For example, it has been observed that IL-4 inhibits the release of pro-inflammatory cytokines, such as IL-6, TNF-α, and NO [89,90].

Analogously to the M1/M2 microglia paradigm, reactive astrocytes have been classified into two phenotypes: A1 and A2 [91]. A1 astrocytes can be activated by cytokines released by M1 microglia [91] and are characterized by the loss of many normal astrocytic functions, such as maintaining synapses [92] and may release a soluble neurotoxin that rapidly kills a subset of neurons and mature oligodendrocytes (OLGs) [91]. In particular, the pro-inflammatory A1 astrocytes increase the expression of different genes (e.g., complement cascade genes) and induce the production of pro-inflammatory factors, such as IL-1β, TNF-α, and NO, contributing to neuroinflammation [88,92]. On the contrary, the A2 phenotype seems to play a neuroprotective role as it induces the synthesis of neurotrophic or anti-inflammatory agents that favor the survival and growth of neurons and support reparative functions [92]. A2 astrocytes may be activated by anti-inflammatory cytokines, such as IL-4, IL-13, and IL-10, and these A2-activated astrocytes may release IL-4, IL-10, and TGF-β [93].

Although the simple dichotomy of the M1/M2 and A1/A2 phenotypes with opposite effects appears to be an oversimplification as activated microglia and reactive astrocytes also exhibit mixed and intermediate phenotypes [94,95,96], it helps in understanding the reactive state of these cells in various CNS disorders.

In conclusion, neuroinflammation is a prominent pathogenic factor for various neurodegenerative diseases such as Parkinson’s disease (PD), Alzheimer’s disease (AD), and amyotrophic lateral sclerosis (ALS) [97,98] and for this reason, represents a target mechanism to neutralize neurodegeneration.

### 3.3. Mitochondrial Dysfunction

The brain is an organ that has a very high demand for energy that can only be satisfied by an appropriate supply of oxygen. The brain requires about 20% of the body’s basal oxygen to perform its function; for example, the Na+ K+ ATPase alone uses about half of the energy produced by the brain to restore the resting membrane potential in the excitatory cells [99]. The high metabolic activity of the brain is mainly supported by the ATP produced during oxidative phosphorylation [100], with cortical neurons being known to consume approximately 4.7 billion ATP molecules per second [101].

Besides the energetic function, mitochondria perform other important functions in the brain, such as the regulation of neurotransmission [102,103] and cell cycle and the control of cell death [104]. Given the importance of these functions, maintaining mitochondrial efficiency is of primary importance [105]. The integrity of mitochondria is ensured by various processes such as biogenesis, fission, fusion, and mitophagy (elimination of damaged mitochondria before they lead to apoptosis of the whole cell) [106,107,108]. Due to the limited regenerative capacity of neurons, mitochondrial dysfunction can have detrimental effects inducing synaptic and neuronal degeneration [109,110,111].

Mitochondria are the main source of ROS production due to the loss of electrons in the electron transport chain [112]. Mitochondrial components are also a direct target of ROS [112], creating a deleterious vicious circle. The iron-sulfur centers of the electron transport chain complexes can be inactivated by acute exposure to ROS, while chronic exposure induces damage to biological macromolecules such as lipids, proteins and DNA [113]. The inner mitochondrial membrane is an extremely vulnerable target of ROS due to its proximity to the production site. ROS can trigger lipid peroxidation, which increases membrane permeability [114], compromising numerous transporters and enzymes and stopping mitochondrial energy production [115,116]. It has recently been shown that damaged mitochondria may also play a role in the inflammatory mechanism by triggering a dangerous signaling response [117,118]. As a result of various stressful stimuli, mitochondrial DNA (mtDNA) can be transported to the extracellular compartment through the generation of extracellular vesicles [119,120,121]. Once released, these vesicles can act as damage-associated molecular patterns (DAMPs) and induce an innate inflammatory immune response by binding to danger signal receptors [117,118].

Several studies show that a mitochondrial dysfunction is an early event in neurodegenerative diseases.

Damage to mitochondria characterized by abnormal mitochondrial morphology, mutations in mitochondrial DNA, altered calcium homeostasis and impaired electron transport chain have been reported in Parkinson’s disease [122]. An Altered mitochondrial morphology, the inhibition of complex IV activity and reduced ATP production have been observed in post-mortem tissues of AD patients [123]. Similarly, in ALS, damage to the mitochondrial structure, dynamics, bioenergetics and calcium homeostasis have been observed [124]. Analyses of blood samples from ALS patients revealed abnormalities in the electron transport chain proteins, including reduced levels of FAD synthase, riboflavin kinase, cytochrome C1 and succinate dehydrogenase complex [125].

Protein aggregates, a hallmark of many neurodegenerative diseases, seem to be associated with mitochondrial damage. Studies on animal and cell models of AD and PD demonstrated that both Aβ and α-synuclein are present in mitochondrial membranes [126,127,128,129]. Furthermore, it appears that α-synuclein reduces the functionality of the electron transport chain complex I, impairing mitochondria and inducing the cell death of dopaminergic neurons [128,130]. Additionally, pathogenic mutations in α-synuclein promote mitochondrial fragmentation, indicating that α-synuclein plays a role in the modulation of mitochondrial morphology [131]. The Tau protein interacts with mitochondrial proteins and alters mitochondrial homeostasis, inducing neurodegeneration and the possible onset of tauopathies [132].

In summary, given the high energy requests of the brain, mitochondria play an essential role in the metabolism of this organ, and therefore, even a negligible dysfunction can have devastating consequences and lead to neurodegeneration.

### 3.4. Oxidative Stress

It is now widely recognized that oxidative stress is responsible for several alterations of the biochemical and biomolecular components that are observed in various neurodegenerative diseases such as AD, ALS, HD, and PD [133]. However, it should be emphasized that low levels of ROS and reactive nitrogen species (RNS) play a fundamental role in the molecular process underlying signal transduction, synaptic plasticity, and memory formation [134,135]. However, when these reactive species are produced in excess, they can damage cellular components such as DNA, proteins and lipids, inducing oxidative stress and triggering neuronal cell death [95]. Oxidative stress can arise both for the reduced activity of non-enzymatic and enzymatic antioxidants or for the excessive production of ROS/RNS. Therefore, a complex balance between oxidant and antioxidant reactions is needed to preserve the functionality of neuronal cells.

Furthermore, as previously pointed out, the human brain is particularly susceptible to oxidative stress, as it uses an enormous amount of oxygen, is highly rich in polyunsaturated fatty acids and redox-active metals (iron, copper), and has low levels of glutathione [136,137].

Mitochondria are mainly responsible for the production of intracellular ROS [138]. From the partial reduction of molecular oxygen in the electron transport chain, the superoxide anion (O_2_^•-^) is formed. Starting from O_2_^•-^, different other ROS are generated, including hydrogen peroxide (H_2_O_2_), hydroxyl radical (HO^•^), hypochlorous acid (HOCl) and the hydroperoxyl radical (HOO^•^) [139]. In mitochondria, the enzyme manganese-superoxide dismutase (Mn-SOD) removes the superoxide, producing hydrogen peroxide, which can be rapidly converted into HO^•^ in the Fenton reaction, catalyzed by the presence of free iron and copper [140]. The hydroxyl radical can also be formed in the Haber–Weiss reaction starting from superoxide anion and hydrogen peroxide [141].

Intracellular ROS can also be produced in the peroxisomes during their oxidative metabolism. The main ROS produced is hydrogen peroxide, whose levels, under physiological conditions, are controlled by the antioxidant enzyme catalase [139].

The production of ROS is the main function of NADPH oxidase (NOX), a multisubunit enzyme complex. Superoxide and hydrogen peroxide are the principal ROS produced [142]. NOX enzymes are expressed in microglia, neurons, astrocytes, and in the neurovascular system. Among CNS cells, there are no reliable data on NOX expression, only in oligodendrocytes [143].

NOX enzymes have an important function in the CNS and are involved in the development, neural stem cell biology, and the function of mature neurons. Among the different isoforms, NOX2 appears to be the most involved in the induction of pathological oxidative stress in the brain, although other NOX isoforms could play an equally important role. Globally, there is now compelling evidence for the role of NOX enzymes in various neurodegenerative diseases such as Alzheimer’s, Parkinson’s, and Huntington’s diseases [144,145,146]. The role of NADPH oxidase in neurodegeneration is further confirmed by the fact that different neurodegenerative diseases seem to benefit from NOX inhibition [143]

Another enzyme responsible for ROS synthesis in the CNS is xanthine oxidase, a flavoprotein that plays a role in purine catabolism by carrying out the oxidative hydroxylation of purines—hypoxanthine and xanthine—to generate uric acid. By-products of these oxidative reactions are ROS, such as superoxide and hydrogen peroxide [147]. Conditions such as Parkinson’s and Alzheimer’s diseases are known to be associated with increased levels of xanthine oxidase activity, potentially contributing to oxidative stress in such conditions [148,149,150].

Nitric oxide synthase (NOS) catalyzes the synthesis of NO^•^ starting from L-arginine and L-citrulline. Although NO^•^ performs important biological functions [151], under oxidative stress conditions, it rapidly reacts with superoxide forming the peroxynitrite anion (ONOO^-^), a highly reactive species. Analogously to ROS, RNS can promote nitrosylation reactions that alter the structure and function of DNA, proteins, and lipids [152].

In the brains of AD patients, there is evidence of ROS-mediated damage documented using different markers for protein, DNA and RNA oxidation as well as lipid peroxidation [153,154]. In the frontal and parietal cortices and in the hippocampus of AD patients’ brains, an increase in protein carbonyl moieties is observed, while proteins are spared in the cerebellum, where no AD pathology occurs [155,156]. An increase in 8-hydroxy-2- deoxyguanosine, a marker of DNA oxidation, was also observed in AD samples with respect to age-matched controls [157,158]. The data on the AD brain are corroborated by studies carried out in transgenic animal models of AD [159,160,161].

In Parkinson’s disease, elevated levels of oxidative stress markers have also been detected, including 8-hydroxy-2-deoxyguanosine, 4-hydroxy-2-nonenal (lipid oxidation) and protein carbonyls and 3-nitro-tyrosine (oxidation of proteins). At the same time, a reduction of the most important endogenous antioxidant, glutathione (GSH), was observed [162].

Higher levels of 8-hydroxy-2-deoxyguanosine, malondialdehyde and lipofuscin (two markers of lipid peroxidation), and 3-nitro-tyrosine were observed in HD brains [163]. Similarly to PD, a reduction in GSH was observed in a HD cell model [164].

## 4. Agri-Food By-Products Targeting Neurodegenerative Mechanisms

**Figure 2:**
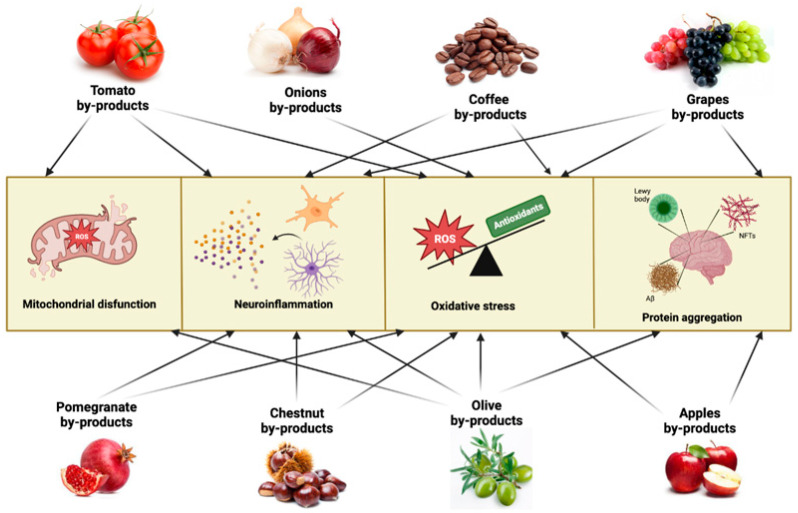
Main neurodegenerative mechanisms targeted by agrifood by-products. ROS—reactive oxygen species, NFTs—neurofibrillary tangles.

Given the multifaced aspect of neurodegenerative pathologies and the difficulties in finding adequate and effective pharmacological treatments, interest is shifting towards the possibility of preventive interventions aimed at contrasting the onset or slowing down the course of pathologies. From this point of view, the use of phytocomponents and/or bioactive molecules of vegetal origin is of great interest, and the possibility of finding these molecules in agrifood by-products and waste represents a common challenge for health and the environment. In this paragraph, we will describe in alphabetical order some agrifood by-products as a potential source to obtain extracts or isolate bioactive molecules capable of counteracting the main mechanisms involved in neurodegenerative pathologies (Figure 2).

### 4.1. Apple By-Products

Apple, a fruit belonging to the Rosaceae family, is one of the most consumed fruits in the world. Specifically, as reported by FAO, apple production has increased by more than a 30% over the past two decades, exceeding 86 million tons in 2020 [165]. Of world production, Italy alone accounted for about 2.5 million tons, which is about 15% of total European production [165]. Annually about 11.6 million tons of the world’s apple production are destined for the production of apple products such as beverages (ciders and juices), sauces, canned and dried apples, and a slice of frozen apple, resulting in the generation of a large amount of waste of about 3.5 million tons/year [17]. Apple pomace is the most representative apple-processing by-product, and its upcycling can be extremely interesting in reducing the environmental impact of food industries and obtaining high-value bioactive compounds. In particular, given its richness in bioactive compounds, apple pomace can be considered for its neuroprotective potential [166].

Apple pomace is the main apple by-product, consisting of peel, stem, seeds, and pulp [17,167] that generate a solid mass that represents about 30% of apple weight. Apple pomace is extremely rich in compounds with high health value, such as dietary fiber, starch, minerals, saccharides, volatile compounds, ursolic acid, and polyphenols [167,168]. For decades, apple pomace has already been exploited to produce animal feed, enzymes, food, textiles, bioethanol, and biopolymers [169,170,171].

In particular, its richness in bioactive compounds can be considered a low-cost source of potential health-beneficial compounds that can be used as functional foods, high-value-added food additives and dietary supplements. Positive effects on the health of apple and apple pomace phytochemicals are well documented. It has been shown that extracts and/or pure compounds isolated from apple pomace are associated with positive effects against chronic-degenerative disorders such as cardiovascular disease, cancer, type 2 diabetes, cognitive decline, and neurodegenerative diseases. In particular, extracts and/or pure compounds derived from apple pomace have shown antioxidant, anti-inflammatory, cardioprotective, antiproliferative, hypolipidemic and hypocholesterolemic activities, antibacterial and antiviral effects, and they also seem to have a positive effect on intestinal microbiota [165,166,167,168,172].

Considering the well-documented neuroprotective effects of phenolic compounds, which are widely present within apple pomace, these apple-processing by-products could be considered a valuable re-upcycling source of bioactive compounds with high neuroprotective potential.

For instance, Alawadi et al. [166] observed that in Sprague-Dawley rats fed with a Western diet calorically substituted with 10% apple pomace, there was a down-regulation of five genes implicated in brain aging and neurodegenerative disorders: synuclein alpha, phospholipase D family member 5, NADH dehydrogenase Fe-S protein 6, choline O-acetyltransferase, and frizzled class receptor 6. In another study, the potential neuroprotective effect of apple pomace crude extract and isoquercitrin (quercetin-3-O-β-D-glucopyranoside) isolated from apple pomace by high-speed countercurrent chromatography was evaluated in an MPTP-induced PD mouse model [172]. Isoquercitrin and crude extract ameliorated the animal behaviors induced by MPTP, reduced the loss of induced dopamine neurons, upregulated tyrosine hydroxylase and dopamine transporters, counteracted apoptosis down-regulating Bax, and reduced MPTP-triggered oxidative stress. This evidence suggests the low-cost reuse of apple pomace and its compounds as promising agents to counteract neurodegeneration.

### 4.2. Chestnut By-Products

The *Castanea sativa Mill.* (chestnut) the tree has long been accorded significant cultural and historical value in mountainous regions for the production of nuts with nutritional properties [173,174,175]. However, the wasteful disposal of chestnut by-products, such as the inner and outer shells (integument and pericarp, respectively) and the spiny burs that surround the fruit and leaves, has always been a global concern. The whole chestnut transformation process is characterized by the production of huge amounts of waste which create serious disposal and environmental problems due to the generation of huge amounts of solid residues that need to be disposed of. In addition, an environmental problem is represented by the habit of burning the shells of chestnuts, activities that may generate several toxic compounds similar to dioxin [176]. Recently, they have been described as interesting sources of added-value compounds with remarkable biological activities [173,177]. Therefore, green-sustainable methodologies should be employed for the recovery of bioactive compounds from chestnut by-products [178,179].

Studies on Castanea sativa by-product extracts ascertained the presence of a large number of phenolic compounds with antioxidant and anti-inflammatory activities, with a potential role in counteracting neurodegenerative diseases [180]. Antioxidant activity and total phenol content of the chestnut inner shell were higher than eucalyptus bark, with a positive linear correlation between antioxidant activity and total phenol content [181]. In addition, the antioxidant activity was linearly positively correlated with the total tannin content, and the main component of the antioxidant activity was hydrolyzable tannin.

For example, *Castanea sativa* shells, an abundant by-product generated during the chestnut peeling process, represent about 20% of the whole weight and are commonly discarded or used as fuel. Recently, several studies demonstrated that *Castanea sativa* shells contain 2.7–5.2% (*w/w*) of polyphenols, and in particular, tannins (condensed and hydrolyzable), phenolic acids (ellagic and gallic acids) and flavonoids (catechin, epicatechin, apigenin, quercetin, and rutin). Polyphenols are generally recognized as interesting bioactive compounds with notable biological activities and pro-healthy properties against oxidative stress-related disorders. Different authors have indicated that tannins are the predominant polyphenolic class in chestnuts [173,181,182]. According to Comandini et al. [183], *Castanea sativa* shells contain about 60% of active tannin substances, including castalagin, castalin, vescalagin, and vescalin, which are easily hydrolyzable. Therefore, they represent an excellent source of molecules with significant biological activities, including antioxidant and anti-inflammatory properties [173,174,184].

Phenolic acids, flavonoids, and hydrolyzable tannins are the main compounds reported in *Castanea sativa* waste [177,184], explaining the beneficial effects exerted by its by-products.

We recently demonstrated that extracts derived from leaves and spiny burs harvested from different *Castanea sativa* cultivars exhibit cytoprotective and anti-inflammatory activity in the LPS-stimulated microglia cell model [185]. Moreover, Isorhamnetin and Kaempferol found in *Castanea sativa* (“Marrone di Roccadaspide”) is involved in counteracting the activation of TLR4, a receptor involved in the neuroinflammation pathway [181].

Cerulli et al. [186] treated a cell line considered as NF-kB/AP-1- Reporter Monocytes stably transfected with MD2/CD14 genes (THP1 and THP-1-XBLUE-MD2-CD14) with extracts of *Castanea sativa* shells or leaves and burs and observed the inhibition of NF-kB activity and NO production after LPS stimulation.

Kang reported the suppression of the excessive NO production and iNOS expression in LPS-stimulated BV-2 cells pre-treated with chestnut (*Castanea crenata*) peel extracts [187].

Moreover, the phenols in the chestnut’s inner shell can improve memory deficits and can be used as a treatment for neurodegenerative diseases. Studies have shown that gallic acid, catechin and epicatechin extracted from the inner shell exhibited positive effects at a preclinical level that may be relevant to the treatment of Alzheimer’s disease and other neurodegenerative diseases [188].

### 4.3. Coffee By-Products

Coffee is one of the most consumed hot beverages all over the world, with over 169 million 60 kg bags of coffee produced in 2020 [189]. Production and consumption of coffee are expected to grow in the next years, and consequently, there will also be an increase in the number of by-products from the coffee industry [189]. In the production of roasted coffee, different by-products are generated, including the husk, pulp, mucilage, parchment, and silver skin, whereas spent coffee grounds are produced either at home or in the industry when the coffee powder is extracted with hot water or brewed to produce instant coffee [190]. The presence of coffee by-products in the environment is associated with several problems due to the different substances they contain, including caffeine, phenols, tannins and organic acids [191]. In particular, the organic matter and other elements present in these agrifood by-products, when disposed of in the soil, can modify the environment, especially of streams and sources, leading, for example, to the death of aquatic beings [192]. Since the disposal of coffee waste is an expensive and complex process, various approaches have been developed to recycle these by-products, including the production of biomass, fertilizers, bioethanol, and fuel in industrial boilers [191,193]. In recent decades, it has been widely demonstrated that coffee has health-promoting potential due to the presence of several bioactive compounds [194]. Coffee beans are rich in such phytochemicals as phenolic compounds, diterpenes (e.g., kahweol and cafestol), triterpenes, methylxanthines (caffeine, theobromine, and theophylline), and trigonelline [195,196].

Several of these bioactive compounds are still present in coffee wastes, and for this reason, they are increasingly being studied as starting material for the production of compounds and extracts to be used as nutraceuticals.

The first by-product obtained during the wet processing of coffee is the pulp, which represents about 30% of the dry weight of the whole bean [197]. Tannins, proteins, carbohydrates, caffeine, chlorogenic acid and total caffeic acid have been identified in the coffee pulp. Coffee cherry husks are a by-product of the dry processing of coffee berries [191]. The coffee husks surround the coffee beans and represent about 12% of the berry on a dry-weight base. Coffee husks mainly contain carbohydrates and, to a lesser extent, proteins and lipids [198]. They are rich in fibers, including cellulose, hemicellulose, and lignin [199].

Coffee silverskin is a thin layer that covers coffee seeds inside the coffee beans and is the unique by-product discarded after the roasting process [200]. It is a residue with a high concentration of soluble dietary fiber (86% of total dietary fiber) and bioactive compounds, such as caffeine, polyphenols and melanoidins [201].

Spent coffee grounds (SCGs) are the residue obtained during the brewing process. The average weight of SCGs constitutes about 75% of the original coffee bean [202]. SCGs is rich in bioactive components such as dietary fiber, polysaccharides, lipids, amino acids, proteins, alkaloids, phenolics, minerals, and volatile compounds [203]. Coffee by-products have been mainly investigated in relation to their antioxidant activity, and only a few of them explored their specific role in counteracting neurodegeneration. Recently, we investigated the neuroprotective effect of SCGs extracts obtained with different solvents (H_2_O, MeOH, a mixture of MeOH:H_2_O (50:50), and a mixture of EtOH:H_2_O (30:70)) in neuron-link SH-SY5Y and BV-2 microglial cells [18]. The extracts were characterized by a high content of caffeine, 5-O-caffeoylquinic acid, 3-O-caffeoylquinic acid, and 3,5-O-dicaffeoylquinic acid. The MeOH extract was the most effective in counteracting oxidative stress induced by H_2_O_2_ in SH-SY5Y through the upregulation of endogenous antioxidant enzymes, such as thioredoxin reductase, heme oxygenase 1, NADPH quinone oxidoreductase, and glutathione reductase [18]. The higher antioxidant capacity of MeOH extract with respect to the other extracts was correlated to a higher content of isogentisin, a compound belonging to the class of xanthones. Interestingly, isogentisin has already been shown to have a potential role in the management of neurodegeneration as it inhibits monoamine oxidase types (MAO) A and B in rat brains [204,205]. MAO-A and MAO-B catalyze the oxidative deamination of endogenous and dietary amines and are both involved in the degradation of serotonin, dopamine, and norepinephrine in the central nervous system [206]. The excessive activity of MAO-A could lead to psychiatric disorders, while the induction of MAO-B seems to play a pivotal role in neurodegenerative diseases such as PD and AD [207,208]. From this point of view, other natural compounds have been demonstrated to inhibit MAO isoforms, such as crocin from Crocus sativus [209] or kaempferol from red wines [210].

On the other hand, H_2_O extract was the most effective in counteracting neuroinflammation induced by lipopolysaccharide in BV-2. This extract led to a strong down-regulation of proinflammatory mediators through the modulation of the TLR4/NF-κB pathway [18]. Of note, no correlation has been found between the anti-inflammatory activity and the presence of specific compounds in the H_2_O extract. In fact, all the characterized compounds were present at a lower concentration in this extract with respect to the other extracts. There is a possibility that the observed anti-inflammatory activity is associated with some bioactive compounds that we have not yet identified.

These data are in agreement with the results of other authors [211,212] that investigated the neuroprotective activity of SCGs extracts obtained with different solvents against the damage induced by two mycotoxins in SH-SY5Y cells, beauvericin and α- zearalenol. Mycotoxins have different deleterious effects on the brain that can lead to neurodegeneration [213,214,215]. Of note, the extract obtained with boiling water was the most effective in counteracting α-Zearalenol-induced damage, while it did not reduce beauvericin -damage [211]. The authors suggested that this different behavior could be associated with some type of interaction between spent coffee constituents, beauvericin mycotoxin, and/or the sensibility of SH-SY5Y. Spent coffee grounds were also used to prepare carbonized coffee bean-derived graphene quantum dots (C-GQDs), a nanodrug candidate for neurodegenerative diseases [216]. Spent coffee beans were dissolved with deionized water before carbonizing them through hydrogen annealing at 1000 °C. Interestingly, C-GQDs showed the ability to inhibit abnormal α-synuclein fibrillation and disaggregate mature α-synuclein fibrils in primary mouse cortical neurons. In addition, C-GQDs cross the blood-brain barrier without significant toxicity [216].

Other studies focused on the general antioxidant activity of SCGs. Andrade C. et al. [189] investigated the in vitro antioxidant activity of spent coffee grounds obtained from different geographical origins (Guatemala, Colombia, Brazil, Timor, and Ethiopia) using the 2,2′ -Azino-bis-(3-ethylbenzothiazoline-6-sulfonic acid) (ABTS) and 2,2-Diphenyl-1-picrylhydrazyl (DPPH) scavenging assays. Even if all the different spent coffee grounds analyzed demonstrated a high antioxidant capacity, the Ethiopian one was the most effective. Interestingly spent coffee ground from Ethiopia was not the richest in polyphenol compounds, so the authors associate this higher antioxidant activity to the presence of non-phenolic compounds in the extracts, not detected in the study. The antioxidant activity of spent coffee grounds and coffee husks extracts, obtained by supercritical fluid extraction (SFE) with CO_2_ and with CO_2_ and co-solvent, was evaluated by DPPH, ABTS and the Folin–Ciocalteau methods [217]. The best antioxidant activity was shown by coffee husk extracts obtained by low-pressure extraction.

Two aqueous extracts obtained by coffee husk and coffee silverskin demonstrated strong anti-inflammatory effects in RAW264.7 macrophage exposed to LPS [218]. In particular, they reduced different inflammatory agents, including iNOS, COX2, the release of NO, PGE_2_, TNF-α (H), and MCP-1. Moreover, they were also able to counteract oxidative stress induced by both LPS and H_2_O_2_ in RAW264.7 cells.

We also demonstrated the antioxidant activity of coffee silverskin extracts in neuron-like SH-SY5Y [201]. In particular, the extracts obtained using different solvents (MeOH, H_2_O, MeOH:H_2_O, EtOH:H_2_O) were used to treat cells before H_2_O_2_ exposure to induce oxidative stress. Extracts obtained with MeOH and EtOH:H_2_O were able to significantly counteract oxidative stress suggesting their potential role in reducing the deleterious effects of ROS in CNS. Coffee silverskin extracts were also evaluated in a neuroblastoma cell line (SH-SY5Y cells) against beauvericin and α-zearalenol-induced cytotoxicity [211,213]. Interestingly, the coffee silverskin extract obtained with boiling water showed the opposite effects compared to the spent coffee extract discussed above; in fact, it was able to reduce beauvericin cytotoxicity but had no effect in counteracting α-zearalenol-induced damage [211].

No studies specifically investigated the neuroprotective activity of coffee pulp, but some of them focused on its antioxidant and anti-inflammatory activity. Magoni et al. [219] demonstrated that EtOH and H_2_O extracts of coffee pulp possess in vitro antioxidant activity (Folin-Ciocalteu and DPPH assays). Moreover, these extracts were able to reduce the release of interleukin-8 induced by TNF-α in human epithelial gastric cells suggesting their potential anti-inflammatory activity. The fresh coffee cherry pulp collected during the crop season 2021 in Thailand was extracted with different methods and was compared in terms of total phenol content (TPC), total flavonoid content (TFC), total tannin content (TTC), and in vitro antioxidant activity by DPPH, ABTS, and FRAP assays [197]. Ultrasound-assisted extraction with propylene glycol (PG-UAE) was significantly higher in TPC, TFC, TTC, DPPH, ABTS, and FRAP response values than UAE with ethanol (EtOH-UAE), maceration with propylene glycol (PG-maceration), and maceration with ethanol (EtOH -maceration). Major bioactive compounds detected included chlorogenic acid, caffeine, and trigonelline. The antioxidant effect of PG-UAE and EtOH-UAE was also investigated in NIH/3T3 fibroblasts exposed to hydrogen peroxide. In the cell system, PG-UAE extract showed a higher ability to counteract oxidative stress than EtOH-UAE. Delgado SR et al. [220] investigated the antioxidant activity of coffee pulps obtained from two varieties of Coffea arabica (var. Caturra and var. Colombia) using two different extraction methods (water or HCl 1%). They observed that acidification of the extraction solvent had an overall positive effect on extraction performance and activity. Moreover, the metabolite concentration and antioxidant activity found in the different extracts were comparable with other agro-industrial residues and even with commercial products.

In conclusion, the different coffee waste products have been mainly studied in terms of antioxidant activity using chemical tests or in vitro cell models. The lack of studies on animal models or even clinical studies will have to be filled in the coming years in order to understand whether these coffee by-products actually play a role in counteracting neurodegeneration.

### 4.4. Grapes By-Products

According to the data from FAO, more than 78 million tons of grapes (*Vitis vinifera*) were produced worldwide in 2020. Italy is the second producer among the leading countries, with more than 8 million tons produced by several appreciated varieties of both red and white wines [165]. Consequently, millions of tons of waste are produced during wine-making processes, causing serious problems for the environment. Developing processes or methods to effectively recover these residues is compulsory, particularly since they are a rich source of bioactive components. Grape pomace or grape marc is a mixture of different grape parts, including seeds, skins, and some parts of the stem [221]. Grape pomace consists of a large number of lipids, dietary fibers (up to 85% depending on the grape variety), minerals, and proteins, but also polyphenols which are mainly retained in the pomace during the production of wine [222]. The grape seeds contain a huge amount of polyphenols like phenolic acids, flavonoids, proanthocyanidins, and resveratrol, while the grape skin is rich in anthocyanins [223]. The presence of polyphenols in wine impacts its taste, color, and flavor, with procyanidins that confer the bitter and astringent taste contributing to the aroma of the wine [224]. The specific phenolic composition of grape pomace can vary depending on cultivars, climate conditions, area of cultivation, and ripening stage of the grapes [225]. Therefore, grape pomace, as well as seeds and skins, represent a massive and low-cost source of natural bioactive compounds with potential beneficial activities on human health. Indeed, polyphenols from grapes possess several biological activities such as anti-atherosclerosis and cardioprotective, neuroprotective, antidiabetic, antioxidant, anti-inflammatory, antiviral, and antimicrobial, which also vary with the grape variety [224].

Large evidence describes the impact of polyphenols isolated from grape pomace (grape seeds and skins) on the nervous system. EGCG inhibited the production of different inflammatory mediators like IL-6, IL-8, and PGE_2,_ and the activation of pro-inflammatory transcriptional factor NF-κB reduced the expression of COX-2 enzyme and the phosphorylation of p38 and JNK MAPKs protecting human U373MG cells from IL-1β + Aβ-induced damage [226]. Epicatechin, kaempferol, and the in vivo metabolite 3′-O-methyl-epicatechin demonstrated to counteract the oxLDL-induced neuronal cell death, possibly through the prevention of the phosphorylation of JNK MAPK and the reduction of caspase-3 activation [227]. As reported in paragraph 4.3, kaempferol from red wines was able to inhibit the hMAO-A isoform, suggesting a potential neuroprotection activity of grape pomace containing this compound [210].

In a hypothalamic cell model, the administration of a water grape extract was demonstrated to counteract the burden of oxidative stress induced by cell exposure to hydrogen peroxide [228]. The whole extract and the catechin alone, as the main component, were able to prevent the increase in the pro-inflammatory COX-2 gene expression after hydrogen peroxide exposure and to restore the levels of BDNF, a crucial neuropeptide correlated to cell survival as well as to learning and memory pathways. Differently, the grape pomace extract, but not catechin alone, halted the reduction in dopamine extracellular levels, indicating that the protective effects can also be ascribed to other phenols present in the whole extract. The grape pomace extract has also been investigated in an oxaliplatin-induced peripheral neuropathy model [229]. Grape pomace extract-treated animals demonstrated ameliorations in the corticospinal functions damaged by oxaliplatin treatment. The protective effect has also been confirmed on the proprioceptive deficits and on all sensorimotor tasks. Grape pomace extract showed to possess anti-inflammatory activity towards lumbar dorsal root ganglions reducing the infiltration of satellite cells after oxaliplatin treatment.

Scola et al. obtained a complex polyphenol mixture from grape seeds of *Vitis labrusca* extract (VLE) cultivated in Brazil [230]. They investigated the potential of VLE as an adjuvant therapy for bipolar disorder, comparing its effects with those mediated by lithium against oxidative damage in SH-SY5Y cells. VLE ensured neuroprotection counteracting the alterations in cell viability and morphology and halting the increase of intracellular calcium and CACNA1c (voltage-dependent calcium channel, L type, alpha 1C subunit) levels.

During the wine-making process, some polyphenols are involved in browning and bitterness reactions, and a clarifying agent like polyvinylpolypyrrolidone (PVPP) is usually added to avoid these modifications. Furthermore, it has been recently described that using PVPP in white wine helps to improve the wine quality and stability by generating a PVPP-white wine extract rich in defined polyphenols [231]. For these reasons, within a context of a circular economy, the potential biological properties of PVPP-white wine extract were explored by Rocha et al. [232]. They evidenced a strong antioxidant capacity of the extract with an EC_50_ of 153.5 µg/mL as a scavenger of superoxide anion. Moreover, the extract’s neuroprotective activity was investigated, and the results demonstrated that it acts as a reversible non-competitive inhibitor towards rat brain AChE activity and protects neuronal cells from glutamate-induced damage and ROS boosting.

As discussed above, grape pomace represents a precious waste residual from wine-making processes because of its richness in polyphenols. These compounds possess many beneficial properties, like anti-inflammatory and antioxidant, useful to counteract neurodegenerative diseases. Unfortunately, these pomaces physiologically undergo extensive degradation after oral administration, dramatically influencing their brain bioavailability. To overcome this obstacle, the researchers are studying several solutions, including novel carrying systems like nutrisomes and an innovative nanovesicle complex [233]. The group of Morelli et al. investigated the effects of pomace extracts from the Nasco grape, a white wine grape cultivar typical of the Sardinia area in Italy, loaded into a nutrisome complex [234]. They compared the biological effects of Nasco pomace-loaded nutrisomes and Nasco suspension in a mouse model of Parkinson’s disease. Interestingly, the results evidenced more significant protection of nigrostriatal dopaminergic neurons using the nutrisome system through the increase in striatal dopaminergic terminals levels and their functionality with respect to Nasco suspension. Other interesting results were obtained by Marino et al. that used nanosized liposomes loaded with a polyphenol-rich white grape extract containing a mixture of different Italian white grape cultivars (60% of Albarola, 30% of Vermentino, and 10% of Bosco) [235]. These extract-loaded liposomes showed to counteract the cellular toxicity of rotenone, a chemical inducer of PD-like features in vitro and in vivo models, in differentiated SH-SY5Y. In particular, the extract-loaded liposomes were able to recover cellular viability, even when added 24 h after rotenone treatment, to decrease the rotenone-induced ROS levels and to completely inhibit the α-synuclein and phospho-α-synuclein aggregation, meanwhile only partially limiting the β3-tubulin depletion. Moreover, the extract-loaded liposomes were demonstrated to pass in adequate concentrations the BBB without affecting the cell performance.

Another important challenge to overcome to make the most of agro-food waste as a source of precious neuroprotective compounds is to develop effective recovery methods that are environmentally friendly and sustainable that boost the extraction output, reducing times and solvent utilization [236].

A new approach for extraction processes from grape pomaces is the utilization of biological extractions using hydrolytic enzymes, like cellulase and pectinase, that hydrolyze structural carbohydrates allowing the recovery of phenols [237]. The study of Rodríguez-Morgado et al. describes the development of an extraction strategy for grape pomaces, consisting of breaking the cell architecture through proteases allowing the solubilization of the protein content [238]. This method led to the recovery of phenols and other molecules with biological activities like peptides, carbohydrates, and lipids in a soluble form. The obtained grape pomace enzymatic extract showed anti-inflammatory activities against LPS-induced inflammation in microglia cells. In particular, the extract reduced the mRNA levels of different pro-inflammatory mediators, including TNF-α, TLR4, IL-1β, Iba1, and iNOS.

Recently, a nutritional supplement has been formulated starting from the Aglianico cultivar grape pomace named Taurisolo^®^ [239]. The most abundant polyphenols present in Taurisolo^®^ were resveratrol, catechins, and their derivatives. The oral administration to male Wistar rats of Taurisolo^®^ showed efficacy against brain ischemia/reperfusion damage, reducing the infarction size, maintaining the microcirculation flux through a massive NO release, and halting the correlated inflammation [240]. Finally, the results from this study suggest that Taurisolo^®^ could be a useful supplement for physiological alteration in brain perfusion during aging.

Grape by-products are among the most studied from an applicative point of view, and some nutritional supplements are already on the market aimed at neuroprotection. It is believed that in the near future, other nutritional supplements will be designed and produced with neuroprotective purposes, given the wide range of molecular targets that the bioactive molecules present in grape by-products have been shown to hit.

### 4.5. Olive By-Products

According to FAO, in the last decade (2011–2020), the annual world average olive production was about 20.9 × 106 tons [241]. The Mediterranean area (mainly represented by Spain, Italy, Greece, and Tunisia) accounts for about 90% of the entire world’s production.

Such a huge olive amount leads to the annual production of about 3.2 × 106 tons of olive oil [242], but it leaves behind an enormous amount of waste and by-products [243]. Olive by-products include waste generated during the olive oil production process, such as pomace, stones, and wastewater [243]. Olive pomace, the solid by-product obtained during 2-phases oil extraction, accounts for about 80% of the entire olive mass [244], while during 3-phases industrial oil extraction, olive mill wastewaters are also produced [243]. Moreover, it is estimated that 18 × 106 tons/year of olive leaves [245] are generated by olive tree crops and the olive harvesting process. Olive waste products, especially wastewater, have long been considered an environmentally impacting material. Nowadays, from a circular economy perspective, numerous studies have been carried out to exploit these waste as biofuels [246,247], animal feed [248,249], and as a source of bioactive compounds with potential applications in the field of health products [243,250,251,252].

The neuroprotective effects of olive oil phenols and the benefits related to olive oil consumption have been well established [4,253,254,255]. However, besides olive oil, olive by-products might represent a source of bioactive compounds with neuroprotective applications. In vitro studies demonstrated that olive seed extract, rich in phenols, exerted an antiapoptotic effect on differentiated neuron-like SH-SY5Y cells exposed to H_2_O_2_ [256]. Olive seed extracts phenolic composition depends, of course, on the cultivar. A comparison among ultrasound-assisted seed extraction from Cobrançosa, Galega, and Picual cultivars demonstrated that the most abundant compounds were: tyrosol, rutin, luteolin-7-glucoside, nüzhenide, oleuropein, ligstroside, with Galega cultivar being the one with the highest total content (14.71 mg Gallic Acid eq./g dry weight) [257]. Moreover, this study showed in SH-SY5Y cell cultures that olive seed extracts exert antineurodegenerative properties by inhibiting acetylcholinesterase (AChE), butyrylcholinesterase (BChE), and tyrosinase (TYR), three enzymes responsible for acetylcholine or L-DOPA degradation [257].

The cultivar, climate, ripeness, and different extraction techniques strongly affect olive mill wastewater extract phenolic content [258,259]. Recently, Gueboudji et al. [260] compared two extraction techniques, liquid–liquid vs. maceration, and demonstrated that dried olive wastewater macerated overnight with methanol allowed to obtain much higher polyphenol extraction (230 vs. 65 mg Gallic Acid eq./g dry weight). Through maceration, 16 different components were detected; among them, those more present were: quinic acid, rutin, hyperoside caffeic acid, luteolin-7-O-glucoside, 4,5-di-O-caffeoylquinic acid, and apigenin-7-O-glucoside. Other studies analyzed olive wastewater phenolic content and also demonstrated the presence of a high amount of hydroxytyrosol [261,262].

Chemical analysis of olive by-products, both pomace and wastewater, demonstrated the presence of hydroxytyrosol esters, namely oleate and stearate, that are not present in olives [263]. It is possible that these esters are synthesized in by-products by esterase activity that couples fatty acids and hydroxytyrosol. However, hydroxytyrosol esters, mainly oleate, exerted in vitro an important anti-inflammatory activity and appeared to be more bioavailable than hydroxytyrosol itself. In fact, the presence of a long fatty acid chain increases the lipophilicity of the entire molecule [263]. Moreover, in SH-SY5Y cells, hydroxytyrosol oleate had a similar antiproliferative effect as hydroxytyrosol but at lower doses [264].

Initial evidence of olive wastewater neuroprotective effects dates back to 2007 [265]. Schaffer et al., in fact, evaluated the possibility of counteracting Fe2+ oxidative and sodium nitroprusside nitrosidative stresses at the murine brain cells level by treating them with hydroxytyrosol-rich olive wastewater extract. Both Fe2+ and sodium nitroprusside induced both ATP and mitochondrial membrane potential loss in ex-vivo brain cells. On the contrary, olive wastewater extract pretreatment allowed for the reduction of ATP loss in both Fe2+ and sodium nitroprusside-exposed cells and membrane potential loss in those exposed to Fe2+. These results suggest olive mill wastewater is a promising candidate for the development of extracts with neuroprotective effects [265].

Pantano et al. [266] compared the effects of 8-week dietary supplementation with oleuropein or a mix of polyphenols extracted from olive wastewater for their ability to counteract the cognitive decline in CRND8 transgenic mice. This transgenic model quickly (2–3 months of age) develops extracellular Aβ deposits. Step down cognitive test revealed that treatments only partially recovered the cognitive decline. However, the Authors demonstrated that both supplementations were able to reduce total plaque area and they both induced autophagy at the cortex level.

Recently, Romero-Márquez et al. [267] evaluated a dry extract from olive fruit containing 20% hydroxytyrosol on Alzheimer’s disease biomarkers by using Cenorabditis elegans as a model system. First, the Authors demonstrated, both in vitro and in their worm model, the strong antioxidant activity of the extract. In fact, it prevented ROS production in worms exposed to oxidative stress induced by 2,20-Azobis(2-methylpropionamidine) dihydrochloride. The in vivo antioxidant effect of the extract was related to its high flavonoid and phenolic content and to the ability to induce SKN-1/NRF2 genes that, in turn, promote antioxidant and detoxifying genes such as SOD-3 and GST-4. SOD-3 and GST-4 are involved both in the counteraction of Aβ plaques deposition and Aβ detoxification. A transgenic nematode model expressing human Aβ at the muscle level showed paralysis as Aβ toxic effect; in this model, the extract counteracted the paralysis, which in turn was not reduced in SKN-1/NRF2 knockout worms. These results lead the Authors to suggest the involvement of SKN-1/NRF2 signaling in the counteraction of Aβ toxicity in Cenorabditis elegans [267].

As previously mentioned, olive tree crops lead to an important accumulation of olive leaves and twigs due to tree pruning and olive harvesting. Even though leaves and twigs are still commonly burned on the field, especially at the small farm level, they can become an interesting source of bioactives to be exploited in the food supplement industries. Olive leaf extracts have been commonly used in traditional medicine for their antimicrobial, hypotensive and cardioprotective properties [268].

Martin-Garcia et al. [269], evaluated the recovery in phenolic compounds from olive leaves by HPLC–MS after the optimization of an ultrasonic probe extraction method. They analyzed the olive leaves extract phenolic composition from 7 different cultivars (Arbequina, Arbosana, Changlot Real, Frantoio, Koroneiki, Picual, and Sikitita). Thirty different phenolic compounds were detected, including oleuropein and hydroxytyrosol. Total phenolic compounds ranged between 28 and 49 mg × g^−1^ of dry matter, and oleuropein (ranging from 14.4 to 34 mg × g^−1^ of dry matter) was the most present in any cultivar analyzed.

The neuroprotective and anti-inflammatory potential of an olive leaves fraction rich in triterpenoid was demonstrated in SH-SY5Y, and Human THP-1 monocytes stimulated with Aβ42 and lipopolysaccharide, respectively [270]. Olive leaves fraction treatment at 40 mg/mL for 24 h revealed anti-inflammatory properties by significantly reducing TNFα, IL-1B and IL-6 secretion in THP-1 cells and counteracted Aβ42 cytotoxicity in SH-SY5Y cells by maintaining cell viability at the same level of control cells. Furthermore, by applying the bioinformatic approach to a lipidomic analysis, the authors demonstrated that their olive leaves fraction treatment significantly increased phosphatidylcholines and phosphatidylethanolamines content in Aβ42 stimulated SH-SY5Y. Phosphatidylcholines and phosphatidylethanolamines have been recently demonstrated to decrease in in vitro Alzheimer’s disease models [271,272], this observation can explain the protective effect of olive leaves triterpenoid against Aβ42 toxicity [270].

Mikami et al. [273] evaluated the neuroprotective potential of an olive leaf extract containing oleuropein and oleanolic acid. Oleanolic acid is a well-known agonist of transmembrane G protein-coupled receptor 5 (TGR5) [274], whose activation induces mitochondrial biogenesis [275], and due to the importance of mitochondria in the maintenance of neuronal function, TGR5 activation has been proposed to counteract cognitive decline. The Authors demonstrated that olive leaf extract administration in mice prevented cognitive declines and decreased depressive behaviors [273].

The olive tree represents a primary source of products useful for human health (just think about the role of olive oil in the Mediterranean diet). Furthermore, the by-products of oil processing and agri-food wastes are becoming increasingly important, given the enormous impact they have on the environment and the high costs associated with their disposal. Therefore, it is important to increase research on these by-products as primary sources of low-cost bioactive molecules with potential neuroprotective efficacy

### 4.6. Onion By-Products

Onions are one of the most widely cultivated and consumed vegetables worldwide. Specifically, as reported by FAO, world onion production was over 100 million tons in 2020, of which Europe accounts for 10% [165]. In the last decade, there has been an increase in onion production of about 24% worldwide and 16.4% in Europe. Italy, with about 460,000 tons per year, is the 8th largest producer of onions in Europe and 38th in the world [17].

Through both domestic consumption and industrial processing, huge amounts of waste are generated each year and generally consist of leather, peels, upper and lower portions and the two outer layers [276].

Although onion waste constitutes a rich source of bioactive compounds, unfortunately, they remain scarcely reused due to their unpleasant and intense aroma, which makes them unsuitable to be used in fertilizers and/or animal feeds [277,278,279]. Onions consist of more than 80% of water and in decreasing amounts of carbohydrates, protein, fat, vitamins, and minerals, respectively [280]. The composition varies depending on the species (pearl, yellow, red or white onions) and cultivation techniques [281,282]. In addition to the macro- and micronutrients already mentioned, onions are extremely rich in phenolic compounds, flavonoids, anthocyanins, flavanols, vanillic acid, tannins, ferulic acid, and sulfur oxides of alkenylcysteine [278,279]. Sulfur compounds are the ones responsible for the particular and distinctive aroma and flavor of onions [278,279]. Of note, most of these compounds are mainly present in onion waste rather than in the edible portion. In fact, it has been shown that most phenolic compounds are found in onion peel/skin [281,282]. The phytochemical composition of waste varies among species. In fact, it was seen that pearl onion peel was the richest in total phenolic compounds, although red onion peel was the richest in flavonoids, especially anthocyanins, which are responsible for the distinctive red/purple coloration [278,279,280,281].

Onion waste, other than being used both to produce natural coloring agents (red onion) [279] and non-toxic nanospheres as pharmacological carriers [283], are particularly interesting for the biological activities associated with their phytoconstituents.

There is increasing interest in the valorization and sustainable use of onion by-products as inputs for the production and formulations of supplements and pharmaceutical preparation, representing a low-cost, high-potential source of health-promoting agents. Several studies investigated the biological activities of onion waste. These by-products have shown antitumor, antimicrobial, cardioprotective, antiobesity, anti-inflammatory, antioxidant, antigenotoxic, antidiabetic, immunomodulator, anti-erectile dysfunction, and neuroprotective properties [278,279,282,284,285,286,287,288,289].

Nile et al. investigated the ability of three extracts (methanolic, ethanolic, and ethyl acetate) obtained from solid onion waste (OSW) to inhibit AChE and BChE, key enzymes in the treatment of Alzheimer’s disease. The activity of these three extracts was compared to three pure flavonols (Quercetin (Q), quercetin-4′-O-monoglucoside (QMG) quercetin-3, 4′-O- diglucoside (QDG)), and galantamine. OSW extracts were shown to be more effective than the reference compounds, suggesting their potential use to counteract neurodegenerative disorders [287]. In another study, the potential antioxidant effects of yellow onion husk ethanolic extract in aging Wistar albino rats were evaluated. Although no effect was found in the plasma and blood, dietary supplementation with the ethanolic extract significantly upregulated the antioxidant enzymes CAT and SOD, suggesting a neuroprotective effect of the extract through an antioxidant mechanism [286].

The effects of the ethyl acetate extract of onion flesh (EOF) and peel (EOP) was investigated in mice treated with Trimethyltin, an in vivo model of cognitive dysfunction [285]. Through Q-TOF UPLC/MS analysis, the two extracts proved to be particularly rich in bioactive compounds such as quercetin and quercetin-4′-glucoside (EOF) and quercetin-4′-glucoside and isorhamnetin-4′-glucoside (EOP). Of course, only the extract obtained by onion peel could be considered a by-product, while the flesh is the edible part of the onion. Both extracts showed a dose-dependent AChE inhibitory capacity in vitro, with a higher efficacy of EOP than EOF. In vivo, they were able to improve learning and memory and increase SOD activity GSH levels and decrease MDA production [288].

In light of these findings, onion waste may be held in high regard as promising neuroprotective agents.

### 4.7. Pomegranate By-Products

The pomegranate is native to Iran, and it is widely grown throughout the world, as it is adaptable to different climatic conditions. Its fruit and juice are considered tasteful and healthy food. The pomegranate peel represents about 40–50% of the total fruit weight, and although it was previously considered waste, it contains numerous and diverse bioactive substances, as recently reported [289,290,291]. Therefore, its recycling not only overcomes the biowaste problems but also provides a source of valuable compounds, such as ellagitannins, flavonoids, and anthocyanins. The extraction technology mostly used are high-pressure-assisted extraction (HPE), acid hydrolysis, and ethyl acetate extraction, pressurized liquid extraction (PLE) [292]. Several studies have been focused on innovative methodology to evaluate how much the growth area, cultivar type, and procedure influenced the chemical composition of the peels extracts in order to concentrate and conserve bioactive compounds with promising biological activities, including but not limited to antioxidant, anti-inflammatory and neuroprotective [289,292,293].

Numerous studies highlighted the presence of a wide variety of phenolic compounds in pomegranate peel, in particular: gallic acid, ellagic acid, catechin, luteolin-7-O-glucoside, rutin, quercetrin-3-O-glucoside, apigenin-7-glucoside, thymol and olivetonide, in addition to the characteristic ellagitannins punicalagin, punicalin, and granatin B. It is noteworthy that Szwaiger et al. discussed the neuroprotective role of phenolic acids from food in a very comprehensive review [294].

Pomegranate peel methanol extract exhibits cholinesterase inhibitory activity in vitro [295] and in vivo, suggesting its potential role in the prevention and co-treatment of Alzheimer’s disease. As far as in vivo experiments are concerned, Amri et al. investigated the beneficial effects of pomegranate extracts (seeds oil, leaves, juice, and peel) in high fat–high fructose diet-induced-obese rat, in terms of brain cholinesterase activity, brain oxidative stress. The results highlight the neuroprotective effects of pomegranate extracts obtained by means of the inhibition of cholinesterase and the increase of antioxidant capacity [296].

In rats with Parkinson’s disease, ellagic acid from pomegranate peel leads to a decrease in MAO-B and an increase in Nrf2 [297].

Moneim showed that the treatment with pomegranate peel could prevent complications associated with aluminum-induced neurotoxicity in rats [298].

Ellagic acid, derived from the ellagitannin Punicalagin, was found to be highly promising in the prevention and treatment of neurodegenerative diseases (including Alzheimer’s disease, and Parkinson’s disease) and brain injury, according to data obtained in preclinical studies [299].The mechanisms underpinning EA activities include not only direct antioxidant and anti-inflammatory activities but also the regulation of the metabolism of neurotransmitters. However, the solubility and intestinal absorption rate of ellagic acid are slow; its metabolism is fast, and consequently, its bioavailability and clinical efficacy are negatively affected. More pharmacokinetic studies should be carried out to develop suitable delivery systems and preparations to improve its bioavailability [300].

In rats with Alzheimer’s disease, ellagic acid from pomegranate peel not only decreases lipid peroxidation but also increases acetylcholinesterase activity and antioxidant enzymes (CAT, GSH, and Nrf2 nuclear/cytoplasmic ratio) and protects hippocampal CA1 pyramidal neurons in a rat model of Alzheimer’s disease. A study showed that EA has a neuroprotective effect by preventing myelin loss in the white matter of the spinal cord [301,302].

In an experimental autoimmune encephalomyelitis, the most common model for multiple sclerosis characterized by inflammatory cell infiltration into the central nervous system and demyelination, Busto et al. showed that the oral administration of ellagic acid delayed the onset and reduced the progression of the disease, as indicated by clinical scores [302].

Shahram et al. treated rats with experimental traumatic brain injury with ellagic acid and found that the treatment (100 mg/kg, IP, every 8 h until 48 h later) can restore the neurological severity score (NSS), cognitive ability, hippocampal function, and enhance the permeability of the blood–brain barrier; furthermore, traumatic brain injury (TBI) results in a significant increase of TNF-α content in brain tissue [303].

Recently, Cásedas et al. [304] demonstrated the neuroprotective role of Urolithin A, a metabolite generated from ellagic acid and ellagitannins by the intestinal microbiota after the ingestion of pomegranate. In particular, this study suggested that urolithin A improves the cellular antioxidant power, acts as a direct radical scavenger and inhibits the activities of oxidase in a cell model (Neuro-2) after oxidative stress induction.

Taken together, experimental evidence suggests that pomegranate peel extract, in particular the one obtained in methanol, can be considered a source of bioactive compounds and helpful in the development of therapeutic drugs for nervous system diseases.

### 4.8. Tomato By-Products

FAO estimates that the world tomato production was about 187 × 106 tons in 2020 [241]. The tomato processing industry is a global leader in the production of huge amounts of solid waste, usually defined as tomato pomace. Such pomace accounts for about 3% (in weight) of the processed fresh tomato [305]. Pomace consists of a mixture of seeds and peels, and it is not suitable for direct human consumption. Most pomace is often discarded as solid waste, or it can be used for animal feed as a feed ingredient [306,307]. However, the chemical analysis of tomato pomace’s composition demonstrated that it could represent a source of bioactive compounds [305].

Of course, different cultivars and ripening levels, climate conditions and soil composition can lead to different contents of such bioactives. However, as reviewed in 2019 by Lu et al. [308], many bioactive compounds have been identified in tomato pomaces. Seeds account for about 60% of the pomace mass [305]; they contain both proteins (up to 40.9 g/100 g) and fats (up to 24.5 g/100 g). Peels, about 40% of pomace [305], contain mainly fiber (up to 88.5 g/100/g), phenolic compounds (157.8 mg (GAE)/100 g), and the carotenoid lycopene (288 mg/100 g). Lycopene exerts a wide spectrum of biological activities, such as antioxidant and anti-inflammatory, resulting in protective effects at multiple tissue levels, such as cardiovascular and brain [309,310].

Moreover, its antiproliferative properties and ability to counteract prostate cancer have been well documented [311]. Solaberrieta et al. [312] recently optimized a microwave-assisted extraction method of tomato seed oil. The oil analysis revealed a high content of palmitate, stearate, oleate, and linoleate. In addition, the presence of γ- and α-tocopherols in the seed oil explained the antioxidant properties of the oil itself. Miklavčič Višnjevec et al. [313] evaluated the presence of bioactive compounds in tomato by-products. They found many different phenolic derivatives belonging to the groups of hydroxycinnamoylquinic acids, flavones, flavanones, flavonols, and dihydrochalcones; among them, caffeic, cryptochlorogenic, protocatechuic, cumaric and chlorogenic acids, rutin, quercetin, and naringenin were found. Overall, these compounds are known for their antioxidant, anti-inflammatory, antiatherogenic, and antiproliferative properties [314].

Kumar et al. [315] extensively reviewed the biological properties of tomato seed extracts and their composition. In fact, tomato seeds also contain significant amounts of more than 30 different phenolic compounds. These bioactives are responsible for different biological properties in vitro and in vivo. Antimicrobial, antioxidant, cardioprotective and antiaggregating activities are among the most well-documented properties [315].

Only a few studies have evaluated the neuroprotective effects of tomato seed extracts. Muralidhara et al. evaluated tomato seed extract’s ability to counteract rotenone-induced oxidative stress and neurotoxicity in both Drosophila and a mouse model [316,317]. In their study on adult male flies, a diet enriched with 0.2% tomato seed extract protected flies against rotenone-induced mortality, oxidative stress, mitochondrial dysfunctions, protein carbonylation, and locomotor impairment. Moreover, the extract improved cholinergic function and dopamine levels [317]. Similarly, in a mouse model, oral administration of tomato seed extract (100 mg/kg b.w. for 3 weeks) alleviated rotenone-induced behavioral phenotype, oxidative stress, mitochondrial dysfunction, and neurotoxicity [316].

Frosini et al. [318] evaluated the effects of an aqueous extract from Lycopersicon esculentum Mill. (“Camone” tomato) in male spontaneously hypertensive rats. The extract was obtained by lyophilizing tomato locular gel and serum, which are rich in phenolic compounds but are often discarded during industrial processing [319]. Treating hypertensive rats with tomato extract for 4 weeks significantly reduced blood pressure. Moreover, ex vivo studies on brain slices revealed that tomato extract protected brain tissue from oxidative stress and reduced the level of inflammatory cytokines.

Tomato peel, as previously mentioned, is an important source of the carotenoid lycopene. Lycopene has been widely studied for its antioxidant, anti-inflammatory, and antiproliferative activities [320,321]. Moreover, due to its lipophilic structure, and if administered as cis-isomers, it can cross the blood–brain barrier and exert its effects at the brain and neuronal levels [322,323]. A wide body of literature investigated lycopene neuroprotective properties, particularly in models of Alzheimer’s diseases; a complete discussion of these aspects is beyond the scope of this review (for a comprehensive review, please see [310,321]).

Tomato waste, therefore, represents an important source of bioactive molecules, attributable not only to the lycopene but to a wide range of potentially exploitable molecules (e.g., phenolic compounds, fatty acids and tocopherols) in the protection of neurodegenerative diseases.

## 5. Discussion

From the analysis of the studies carried out on waste products taken into consideration in this review, it emerges that, to date, only preclinical studies have been conducted, and most of them are on cell cultures (Table 1). Among the various by-products, those obtained from pomegranate have the largest number of “in vivo” studies on animal models. The lack of clinical studies, therefore, does not allow us to state with certainty that these by-products can be effective in humans in preventing or treating neurodegeneration. With the knowledge we currently have, we can only conclude that these waste products have enormous potential as starting materials to develop products for the treatment of neurodegenerative diseases. The future development of three-dimensional cellular models could help to link “in vitro” models and “in vivo” models and help to obtain results in the identification of bioactive molecules able to slow down the progression of the disease. Despite the beneficial consequences of the different extracts or compounds obtained from the by-products analyzed in this review, another aspect to consider is their bioavailability which could be a limitation to the study of their clinical applications. After dietary consumption, phytochemicals undergo an intense metabolism; they can be modified by the pH of the stomach, by the metabolism of the intestinal microbiota, they cannot be taken up by the intestinal absorptive cells and, if absorbed, they are subjected to phase II metabolism in the liver.

Furthermore, considering that we are focusing on neurodegeneration, further criticism is represented by the high selectivity of the blood–brain barrier. To increase the bioavailability of these preparations from plant-origin by-products the use of technological formulations should be developed. Nanoencapsulation could extend circulation, improve localization, boost efficacy, and lower the risk of multidrug resistance [324]. Some authors are already exploring this aspect and, as reported above, to increase the bioavailability of a pomace extract from grapes, Parekh et al. [234] loaded them in nutrisomes, an innovative nanovesicle complex.

Another important aspect to consider is the role played by the solvents used to produce the different by-product extracts. Among the most used solvents are ethanol, methanol, water, propylene glycol, ethyl acetate, or various mixtures of these solvents. The biological effects of the various waste product extracts seem to be strongly linked to the type of solvent used. For example, we have demonstrated that an SCG extract obtained in methyl alcohol was effective in counteracting oxidative stress in neuron cultures, while the extract prepared in water did not show any antioxidant activity [18]. In addition to the impact of the solvent on the potential biological activity, it must be taken into consideration that the production of these products must be sustainable and therefore have a minimum impact on the environment. From this point of view, the use of green solvents represents a fundamental and essential approach. To respond to this need and to overcome the limitations associated with conventional extraction methods, new extraction techniques have been developed in recent years. Among the most cutting-edge technologies are pressurized liquid extraction, supercritical fluid extraction, ultrasound-assisted extraction, deep eutectic solvent-assisted extraction, cold plasma-assisted extraction, microwave-assisted, enzyme-assisted extraction, and electrical technologies [325]. These “green” extraction techniques have many advantages, including less time, energy and solvents and are therefore in line with sustainable development strategies. Furthermore, the use of “green” solvents leads to the production of chemical-free products recognized as safe, a fundamental aspect of human consumption. Another important step in the development of nutraceuticals from agrifood by-products is the standardization of the preparation. Unfortunately, the same waste material can come from different sources, and its reuse is considered secondary and unimportant, so no attention is paid to its production. To overcome this problem, it will be necessary to develop analytical techniques that will allow quick and cheap characterization of the different products obtained.

Of note, compared to the product that generated them, waste can have an added value, like in the case of pomace and wastewater obtained by olive oil production. This waste containing hydroxytyrosol esters, which are not present in olives, was demonstrated to be more effective than hydroxytyrosol [263].

Finally, it is important to note that the utilization of nutraceuticals targeting neurodegeneration obtained by cheap and highly available agrifood wastes offers an invaluable resource for developing new potential therapeutic weapons for combating these devastating diseases. This could be of great importance bearing in mind that these pathologies mainly affect the elderly, who are also those who have fewer economic resources. Moreover, effective health interventions focused on prevention programmes could decrease the economic impact by ensuring that people can age healthfully. Such interventions targeted in midlife could represent an incentive for people who can expect to live long to invest when younger. Furthermore, in countries such as Italy, where public health covers all or part of the costs for treatment, having low-cost compounds available to treat pathologies represents a highly desirable objective with an enormous impact on the economy of the whole country. Clearly, these are long-term aspirational goals; however, in our opinion, the scientific community should pay more attention to the huge potential benefits to the global population and the environment. Fortunately, some products obtained from agrifood wastes are already on the market, such as the already mentioned Taurisolo© obtained from grape pomace.

We can conclude that extraction can be considered a stepping stone in the development of nutraceuticals from agrifood by-products, and this will probably be the most promising lead to follow. On the basis of the evidence reported in the literature for plant-derived neuroprotective agents, further research on the possibility to re-utilize waste and to evaluate their neuroprotective efficacy, especially on human subjects, is well warranted.

## 6. Conclusions

Agricultural waste can be produced at different stages of production, from harvesting to processing, and they are mainly represented by fruit and vegetable by-products in the form of seed, pulp, or peels. These are often managed by uncontrolled disposal via open dumping or burning, with considerable environmental risk. Since the global population and the food demand will continue to rise, resulting in an increase in agrifood waste, it is crucial that our agricultural model will shift towards a totally sustainable one. To face this green challenge, a different concept is in demand—not “waste as a problem” but “waste as resource” by reintroducing agricultural by-products into the circular economy as novel and higher added value compounds. This next-generation revalorization, known as upcycling, may have important repercussions for health industries, promoting the sustainable development of the agro-food chain with environmental and economic benefits and enhancing competitiveness for the industries.

In this context, the development of new bioanalytical/biochemical approaches for the recovery and extraction of bioactive molecules in agrifood by-products and for the characterization of their biological activities is necessary, above all in the field of neurodegenerative diseases, which will affect an ever-increasing number of subjects in the world in the future

## Figures and Tables

**Table 1:** List of the biological effects of the different by-products.

Plant	By-Product	Biological Activity	Model System	Ref.
Apple	Pomace	Down-regulation of synuclein alpha, phospholipase D family member 5, NADH dehydrogenase Fe-S protein 6, choline O-acetyltransferase, and frizzled class receptor-6 genes	Sprague-Dawley rats fed with a Western diet calorically substituted with 10% apple pomace	[166]
		Antioxidant; antiapoptotic, upregulation of tyrosine hydroxylase and dopamine transporter, reduction of neuronal loss	MPTP-induced PD mouse model	[172].
Chestnut	Shell	Antioxidant and anti-inflammatory	Macrophages	[183]
	Leaves and Spiny Burs	Antioxidant and anti-inflammatory	LPS-stimulated BV-2 microglial cells	[185]
	Shell, Leaves and Spiny Burs	Anti-inflammatory	NF-kB/AP-1- Reporter Monocytes stably transfected with MD2/CD14 genes stressed by LPS	[186]
	Peel	Anti-inflammatory	LPS-stimulated BV-2 microglial cells	[187]
Coffee	Spent coffee ground	Antioxidant	H_2_O_2_-treated SH-SY5Y	[18]
		Anti-neuroinflammatory	LPS-treated BV-2 microglial cells	[18]
		Protection against mycotoxins	SH-SY5Y	[211,212]
		Inhibition of α-synuclein aggregation	primary mouse cortical neurons	[216]
		Antioxidant activity	In vitro	[187,216]
	Husk	Antioxidant and anti-inflammatory	LPS-treated RAW264.7	[219]
	Silverskin	Antioxidant and anti-inflammatory	LPS-treated RAW264.7	[219]
		Antioxidant	H_2_O_2_-treated SH-SY5Y	[201]
		Protection against mycotoxins	SH-SY5Y	[211].
	Pulp	Antioxidant activity	In vitro	[197,219,220]
		Anti-inflammatory	Human epithelial gastric cells	[219]
		Antioxidant	H_2_O_2_-treated NIH/3T3 fibroblasts	[197]
Grapes	Pomace	Antioxidant, anti-inflammatory with an effect on BDNF and dopamine levels	H_2_O_2_-treated HypoE22 cells	[228]
		Anti-inflammatory; reduction of proprioceptive deficits and amelioration in the corticospinal functions	Oxaliplatin-induced neuropathy in rats	[229]
		Anti-inflammatory	LPS-treated N13 microglia cells	[238]
		Protection of dopaminergic neurons	MPTP mouse model of Parkinson’s Disease	[234]
		Antioxidant; inhibition of α-synuclein and phospho-α-synuclein aggregation	Rotenone-treated differentiated SH-SY5Y cells	[235]
		Anti-inflammatory; reduction of infarction size, maintenance of microcirculation flux	Ischemia/reperfusion rat model	[240]
	Seeds	Antioxidant and neuroprotective	H_2_O_2_-treated SH-SY5Y cells	[230]
	PVPP winery residue	Antioxidant and AChE’s reversible non-competitive inhibitor	Glutamate-treated SH-SY5Y cells	[232]
Olive	Seed	Antiapoptotic	H_2_O_2_-treated SH-SY5Y	[256]
		Inhibition of AChE, BChE, and TYR	SH-SY5Y	[257]
	Wastewater	Reduction of mitochondrial dysfunction	Fe2+ and sodium nitroprusside exposed murine brain cells	[265]
		Reduction of Aβ deposits, induction of autophagy	CRND8 transgenic mice	[266]
	Leaves	Reduction of Aβ42 toxicity	Aβ42-treated SH-SY5Y	[270]
		Anti-inflammatory	LPS-treated human THP-1 monocytes	[270]
		Prevention of cognitive declines and reduction of depressive behaviors	C57BL/6J mice	[273]
Onion	Solid waste	Inhibition of AChE and BChE	In vitro	[287]
	Husk	Antioxidant	ageing Wistar albino	[286]
	Flesh and peel	Antioxidant; inhibition of AChE	Trimethyltin-induced cognitive dysfunction mice	[288]
Pomegranate	Peel	Antioxidant	Neuro-2 cells	[295]
		Antioxidant and anti-inflammatory	Rat Parkinson’s disease model	[302]
		Antioxidant and anti-inflammatory	Rat Alzheimer’s disease model	[301,302]
		Antioxidant	Rats with aluminum-induced neurotoxicity	[298]
		Antioxidant and anti-inflammatory	Rats with experimental traumatic brain injury	[303]
	Seeds oil, leaves, juice and peel	Antioxidant and anti-inflammatory	High-fat-high fructose diet induced-obese rat	[296]
		Anti-inflammatory	Autoimmune encephalomyelitis cell model in Lewis rats	[302]
Tomato	Seed	Antioxidant	Rotenone-exposed male D. melanogaster	[317]
		Antioxidant	Rotenone-exposed male mice	[316]
	Tomato locular gel and serum extract	Antioxidant ad anti-inflammatory	Brain slices from male spontaneously hypertensive rats	[318]

## Data Availability

Not applicable.
